# Supplementary material for: “Younger women had more access to COVID-19 information”: An intersectional analysis of factors influencing women and girls’ access to COVID-19 information in Rohingya and host communities in Bangladesh
Source: PLOS Glob Public Health. 2022 Dec 5;2(12):e0000459. doi: 10.1371/journal.pgph.0000459 (PMC10022011; doi:10.1371/journal.pgph.0000459)
Supplement: Attachment — Submitted filename: Response to Reviewers.docx [file pgph.0000459.s001.docx]

The Editor

PLOS Global Public Health

Dated: 3-8-2022

Subject: Rebuttal letter

Dear Editor,

We are pleased to share the rebuttal letter with responses to the reviewers’ comments for the manuscript ID PGPH-D-22-00607, titled “Younger women had more access to COVID-19 information”: An Intersectional Analysis of Factors Influencing Women and Girls' Access to COVID-19 Information in Rohingya and Host Communities in Bangladesh.

With this, we assure you that we have incorporated all the feedback shared by the respective reviewers and appreciate their time, effort and intellect that has reinforced the manuscript and made it strong.

| S.No | Comment | Response |
| --- | --- | --- |
| Journal-related comments | | |
| 1. | Please provide additional details regarding participant consent. In the ethics statement in the Methods and online submission information, please ensure that you have specified whether:  1) whether the ethics committee approved the verbal/oral consent procedure,  2) why written consent could not be obtained, and  3) how verbal/oral consent was recorded. If your study included minors, please state whether you obtained consent from parents or guardians in these cases. If the need for consent was waived by the ethics committee, please include this information. | Thank you for the comment.  The verbal consent approval has been updated as recommended (Manuscript Line # 270 – 275).  The justification for not obtaining written consent has also been updated. (Manuscript Line # 270 – 275).  The verbal consent procedure has also been updated. (Manuscript Line # 274-277).  In the case of adolescents, the consent was taken either from their parents or guardians AND the verbal assent was taken from the adolescent participants. This statement has been updated in the manuscript. (Manuscript Line # 270 – 272). |
| 2. | Please update the Funding Information in the system to matched with the Financial Disclosure Statement. | The funding information has been updated to match with the Financial Disclosure Statement. (Manuscript Line # 952 – 957) |
| 3. | "Please provide separate figure files in .tif or .eps format and removed from the manuscript file. | The Figures have been removed from the manuscript and have been uploaded separately as .tif format. |
| 4. | Please update the 'Competing Interests' statement with this "The authors have declared that no competing interests exist". | The Competing interests statement has been updated. (Manuscript Line # 958 – 959). |
| 5. | Please review your reference list to ensure that it is complete and correct. If you have cited papers that have been retracted, please include the rationale for doing so in the manuscript text, or remove these references and replace them with relevant current references. Any changes to the reference list should be mentioned in the rebuttal letter that accompanies your revised manuscript. If you need to cite a retracted article, indicate the article’s retracted status in the References list and also include a citation and full reference for the retraction notice. | The references have been reviewed and are correct and meet the guidelines of the Journal. |
| Comments to the Author – Reviewer 1 | | |
| 1. | The introduction needs to be expanded - while it makes it clear what will be discussed in the paper, it falls short of providing a sufficient overview before delving into the topic. For readers that are entirely unaware of the context, a bit more needs to be presented there. | Thank you for the suggestion. More details on the context have been added as per the suggestions of the reviewer. (Manuscript Line # 107 – 127). |
| 2. | The introduction is also recommended to conclude with the main research question. Although this is clear in the title, it still needs to be clearly reformulated in the text) | Thank you for the suggestion. The introduction section now concludes with the main objective of the study. (Manuscript Line # 226 – 228). |
| Comments to the Author – Reviewer 2 | | |
| 1. | Explanation of purda (line 132 and line 313) has been repeated twice. Authors can remove the repetition. | Thank you for the remark. The repetition has now been removed. (Manuscript Line # 338). |
| 2. | The findings presented from line 436 to the end of that section match better with socio-cultural belief section. Because those lines discussed the fatalistic religious belief hampered access to information. | Thank you for the suggestion. The paragraph has been moved to the socio-cultural belief section. (Manuscript Line # 524 – 533). |
| 3. | Some points discussed in power and privilege section, (from line 569) in my opinion, would better match in the socio-economic background section, particularly those cases where school dropout and early marriage happened due to job loss of parents. In fact, power and privilege section does not properly reflect that people like NGO workers are holding ‘power’ for which they are getting extra advantage. They are in ‘privileged’ position though, as findings suggest. The authors may rethink about the word ‘power’ here. | Thank you for the suggestion. The explanation of “power” has been added in the said section. (Manuscript Line # 575 – 577).  The noted paragraph has also been rephrased to better reflect the power dynamics. (Manuscript Line # 607 – 617). |
| 4. | It looks like a word is missing in line 365 | Thank you for the remark. The missing word has been added. (Manuscript Line # 389). |
| 5. | Some typos need to be checked. | Thank you for the comment. The paper has been checked for typos and all the necessary corrections/additions have been made. |

We are hopeful that our manuscript will now be deemed fit for publication.

Best regards,

Ateeb Ahmad Parray

Corresponding Author
